# Identifying the personal characteristics of decent work perception for nursing students in China using latent profile analysis

**DOI:** 10.1186/s12909-024-05206-7

**Published:** 2024-03-20

**Authors:** Ruijing Wang, Haixia Yu, Xuanye Han, Yang Yang, Dong Chen, Qichao Niu, Yanhua Liu, Mingzhu Zhou, Xinyu Zhu, Yuhuan Zhang

**Affiliations:** 1 Second Affiliated Hospital of Harbin Medical University, Prenatal Diagnostic Center, Harbin, Heilongjiang Province 150000 China; 2 Second Affiliated Hospital of Harbin Medical University, Harbin, Heilongjiang Province 150000 China; 3 Department of Neurosurgery, Second Affiliated Hospital of Harbin Medical University, Harbin, 150000 China; 4 Second Affiliated Hospital of Harbin Medical University, Harbin, Heilongjiang Province 150000 China; 5 Nursing General Teaching and Research Department, Heilongjiang Nursing College, Harbin, Heilongjiang Province 150000 China; 6 Student Work Department, Research Institute, Second Affiliated Hospital of Harbin Medical University, Harbin, Heilongjiang Province 150000 China; 7 Second Affiliated Hospital of Harbin Medical University, Student Work Department, Harbin, Heilongjiang Province 150000 China; 8 Research Department, Second Hospital of Harbin Medical University, Harbin, Heilongjiang Province 150000 China

**Keywords:** Nursing students, Decent work;perception, Latent profile analysis, Characteristics

## Abstract

**Background:**

Given the importance of perceptions of decent work for nursing students' future career choices, we attempted to determine potential classifications and characteristics of nursing students' perceptions of decent work so that targeted interventions could be developed.

**Methods:**

A convenience sample of 1004 s- to fourth-year nursing students completed the General Information Questionnaire, Self-Regulatory Fatigue Scale, Occupational Identity Questionnaire, and Decent Work Perceptions Scale in a cross-sectional survey in Heilongjiang Province, China, resulting in 630 valid questionnaires with a valid return rate of 62.75%. Nursing students' perceptions of decent work were defined using descriptive and regression analysis.

**Results:**

Latent profile analysis (LPA) identified three subgroups: low perceived decent work group, medium perceived decent work group, and high perceived decent work group, accounting for 4.76%, 69.37%, and 25.87% of the sample, respectively. The results of unordered multiclass logistic regression show that nursing students with relatively low levels of perceived decent work are more likely to have a low professional identity, a lack of respect for nursing seniors, an involuntary choice of nursing major, and a low family income.

**Conclusion:**

Different types of nursing students have different perceptions of decent work, and these universities and related departments can use different educational guidance strategies.

## Introduction

Work is a crucial aspect of life and a means of expressing one's self-concept. Work is used by people to maintain their sense of self-worth and interpersonal relationships, as well as to achieve better physical and mental health [1]. On the plus side, at least, work is a significant source of income for both consumption and savings. It serves as a social anchor that may offer a sense of worth and significance [2].

The International Labor Organization developed the concept of decent work as a conception of employment quality that expresses the right of all people to decent and productive work in conditions of freedom, equity, security, dignity, and opportunities for personal development and social integration [3, 4]. Existing research indicates that decent work promotes positive outcomes such as adequate remuneration for work, a sense of stability in social relationships, adequate time and rest, and congruence between family and work values [5, 6]. The psychological theory of work argues that decent work is a central factor in individual career choice and development, highlighting not only the objective level of requirements but also the subjective perception of workers and the need for workers to achieve self-worth and dignity on a psychological level [7, 8].

The perception of decent work is a psychological assessment of whether or not workers are obtaining or enjoying decent work, including work rights, wages, work environment, working hours, and other aspects of decency [9]. It can reflect the extent to which an individual perceives work reward, job position, career development, career recognition, and work environment [10]. Hu et al. [11] note that the perception of decent work is primarily based on the psychological experience of workers and the value and self-esteem of workers as a result of valuing their work's meaning. Burchell et al. [12] stated that, from a job satisfaction standpoint, "decent work perceptions" can also be understood as people's job expectations and perceptions of the quality of their work. Numerous researchers have examined the relationships between decent work and happiness [13], positive self [14], psychological contract [15], work engagement, work motivation, and burnout [16]. Some researchers have also suggested that decent work can accurately predict employee motivation, creativity, and performance [17, 18]. Consequently, many academics are focusing more on career education and counseling from the perspective of decent work perception [19].

Due to China's large geriatric population, high prevalence of chronic diseases, and escalating demand for care, the issue of inadequate allocation of nursing human resources is gaining prominence. As a key conduit for delivering clinical nurses to social positions, the nursing profession plays an undeniable role in talent cultivation, career identity, and career choice in this context.

Chinese nursing students (henceforth "nursing students") are the integral and essential reserve force for the development of the nursing profession and the military leaders in the nursing field. According to studies, the perception of decent work has a significant impact on individuals' career choices and is a crucial component of career education and career guidance [20]. It is obvious that nursing students' perceptions of decent work may influence their future career choices and, in turn, the future development of the nursing profession.

However, no research has examined nursing students' perceptions of decent work. Existing studies demonstrate that people may perceive decent work differently, which could lead to the existence of potential subgroups of decent work in diverse populations [21]. However, few studies have examined the heterogeneity of population perceptions of decent work among nursing students. Therefore, the purpose of this study was to determine (1) how the potential analysis was categorized based on nursing students' perceptions of future decent work and (2) the factors that contribute to these disparities.

Latent profile analysis (LPA) is an individual-centered analysis technique that can better distinguish group categories by grouping individuals with similar response patterns into the same subgroup and elucidating response characteristics and the proportion of individuals on different entries or dimensions [22]. Latent profile analysis has been widely utilized in numerous studies and is regarded as having strong scientific and persuasive power in identifying subgroup classification [23, 24], and [25]. This study aims to investigate the potential categories of nursing students' perceptions of decent work and the differences in their characteristics using the latent profile analysis approach with the goal of providing a reference for career guidance in universities and related departments.

### Objects and methods

In Heilongjiang Province, China, we commenced a cross-sectional study at two universities. Select students from two institutions in Heilongjiang Province using a convenient sampling technique. One university is a vocational college with a vocational education level, and junior college is the highest level of education that its graduates have attained, and they need to complete 3 years of study. The number of students in the school is about 7,000.The other institution is an ordinary university, which belongs to the undergraduate level and above education and can train both three-year junior college students (nursing students need to complete 3 years of study) and four-year undergraduates (nursing students need to complete 4 years of study), and the number of students in the school is about 1,200. Using online structured questionnaires (https://www.wjx.cn) to collect data from second- to fourth-year nursing students at these two universities and then developing and testing a potential cross-sectional model of Chinese nursing students' perceptions of prospective decent work.

### Subjects

The inclusion criteria for participants in this study are as follows: (1) registered professional nursing students or undergraduate nursing students; (2) nursing students in their sophomore to senior years (i.e., with more than 2 years of study); (3) students who understand the purpose of this study; (4) refers to students who voluntarily participate. Exclusion criteria: nursing students who are unable to participate in the survey due to school leave, personal leave, or sick leave.

Here, we would like to point out that, before the start of our research, we searched a large number of relevant studies to ensure the comprehensiveness and scientificity of the inclusion of demographic variables in our research. But research suggests that, due to the limited time that first-year nursing students spend studying nursing, their understanding of the nursing profession is shallow, cognitive bias exists, and they believe that nursing cannot fulfill their life goals [26]. Therefore, in the selection of the population, considering that this portion of the population is prone to having some bias in the assessment of decent work perception, nursing students in their freshman year are excluded. We also found that studies have confirmed that age has no significant impact on decent work for nursing students [27, 28]. As a result, our study did not include age variables for students. Pay more attention to the impact of factors such as clinical internship time, school activities, and role model strength on students' perceptions of decent work.

According to Kendall's sample estimation method [29], the sample size for cross-sectional survey studies must be at least five to ten times the observed variables plus twenty percent sample attrition. This study included a total of 81 submissions, and the calculated sample size was at least 486. In addition, Sinha [30] suggested that latent profile analysis can be considered a "large sample" technique, but the accuracy of the model and fitted statistics is consistently high when the sample size exceeds 500. And therefore, the sample size for this study is sufficient.

### Data collection method

The researchers implemented the questionnaire on the Questionnaire Star platform (https://www.wjx.cn/vj/PRuTQS0.aspx). At the beginning of the question–answer interface, participants are clearly informed that the data is only used for academic research, and an anonymous filling system is adopted to ensure that participants truthfully fill out the questionnaire. Before the survey began, six nursing students were randomly selected for a preliminary survey. The questionnaire measurement time was around 5–10 min, and each item was reasonable. The questionnaire is set to be submitted once and cannot be answered twice. In the end, 1004 questionnaires were collected, 630 valid questionnaires were recovered, and the recovery rate was 62.75 percent. The exclusion criteria for invalid questionnaires were: 1) questionnaires with obvious regularity of answers, like choosing the same option for all entries; 2) questionnaires with inadequate responses; 3) questionnaires in which each response is submitted within three minutes; 4) questionnaires in which responses contain logical contradictions; and 5) excluding freshmen and non-registered students who fill out questionnaires.

The survey was conducted from November 1 to November 20, 2022. We invited a teacher as a liaison at each of the two universities, and the questionnaire link was sent to the students through the liaison. We also requested that their student ID be provided as a unique identifier in the survey. After students see the link, they can directly click on the link according to their true intention to enter the question-answering interface. They can complete anonymous forms according to their actual situation and submit the results directly to the online website backend. The collected data is handed over to the data management personnel of our research group, and other personnel have no right to view it.

### Survey tools

#### General Information Questionnaire

There are 10 things to look at: gender, education level, grade, whether or not they are an only child, where they were born, how often they take the initiative to join different activities put on by the college every month, whether or not they chose nursing as their major, how long they spent in clinical practice, whether or not they looked up to nursing seniors, and the average monthly family income (in yuan). The various activities organized by the college, including practical skills competitions, 512 Nurses' Day, small lectures, etc., help to enhance students' professional awareness and promote personal professional abilities. Involuntary choice of nursing major: forced to transfer to nursing major due to unsatisfactory college entrance examination results.

#### Self-Regulatory Fatigue Scale

The scale was created by Canadian scholar Nes et al. [31] in 2013 and translated into Chinese by Wang Ligang et al. [32] in 2015. It has 16 entries,including three dimensions of cognitive control, emotional control, and behavioral control. Representative items include feeling energized, being able to easily set goals, and having difficulty executing my exercise plan. The questionnaire is scored at 5 points, with 1=strongly disagree; 2= disagree; 3=uncertain; 4= agree; and 5= strongly agree. Among them, items 1, 2, 5, 9, 11, and 14 are reverse scoring. for a total score of 30 for the cognitive control dimension, 25 for the emotional control dimension, and 25 for the behavioral control dimension. All of the items were added up to get a total score, and high scores showed that self-regulation fatigue was very bad. In this study, the Cronbach's alpha coefficient was 0.850, the KMO value was 0.894, and the approximate value of Barlett's sphericity test was x^2^ = 5428.001, *p* ＜ 0.001. This means that the study was reliable and valid.

#### Occupational Identity Questionnaire

Hao Yufang [33] The questionnaire contains five dimensions and 17 items. The 5 dimensions are career self-concept, benefits of staying and risks of leaving, social comparison and self-reflection, freedom of career choice, and social persuasion. For example, I am willing to become a nurse; I will not change my current career direction; I enjoy my profession; and I am prepared to develop in this direction. The questionnaire adopts the Likert 5-level scoring method, with "very non-compliant = 1 point", "less compliant = 2 points", between = 3 points", "more compliant = 4 points", and "very compliant = 5 points. The total score ranged from 17 to 85, with a higher score indicating a stronger occupational identity. In this study, the Cronbach's alpha coefficient was 0.950, the KMO value was 0.965, and the approximate value of Barlett's spherical test was x^2^ = 10144.038, *p* ＜ 0.001. This means that the study was reliable and valid.

#### Decent Work Perception Scale

This scale was made by Mao Guanfeng et al. [34]. So that intern nursing students could use the survey more easily, the entries were changed from the present to the future tense. For example, "My workplace can provide me with generous benefits" became "My future workplace can provide me with generous benefits. This scale includes five dimensions: job return, job position, career development, career recognition, and work atmosphere, for a total of 16 items. The Likert 5-level scoring method is used, with completely disagree = 1, less agree = 2, between = 3, basic agree = 4, fully agree = 5.Among them, the job position dimension is scored in reverse (representing items such as: I anticipate that the workload in the future will be so high that I will often be overloaded). I anticipate that the workplace environment will be crowded, noisy, or dull in the future. I anticipate that I will work overtime frequently in the future. The total score of the scale is 16–80 points, and the higher the score, the higher the nurse's perceived level of decent work. In this study, the Cronbach's alpha coefficient was 0.824, the KMO value was 0.895, and the approximate value of Barlett's spherical test was x^2^ = 5686.836, *p* ＜ 0.001, with good reliability and validity.

### Statistical methods

SPSS 21.0 and Mplus 7.4 were utilized for data analysis and statistics. Mplus was used to undertake a category analysis of nursing students' opinions of decent work. LPA was applied to analyses in which the exogenous variables contained continuous data. Using the latent profile analysis module of Mplus 7.0 software, the data were evaluated, and the model fit test indications [18] included: (1) log likelihood (LL)、Akaike information criterion (AIC)、Bayesian information criterion (BIC)、adjusted Bayesian information criterion (aBIC), and the model fits better the smaller the aforementioned four parameters. The above four values do not have critical values, and the smaller the value, the better the fit of the model. (2) The value of entropy, which runs from 0 to 1. The closer to 1 the categorization accuracy is, the greater it is. (3) Lo Mandell Rubin (LMR) and the Bootstrap Likelihood Ratio Test (BLRT), are used to evaluate the fitting differences of the latent profile model. When *P* < 0.05, the model representing K categories is significantly better than the model representing K-1 categories [30]. The optimal model needs to meet the following criteria: AIC, BIC, and aBIC values are the smallest in the model; entropy values are > 0.7; and LMR and BLRT are *P* < 0.05.

This study used the five-dimensional items of the Decent Work Perception Scale as indicators to perform classification model fitting estimation on nursing students' decent work perception. Starting from a single-category baseline model, establish a data model, gradually increase the number of categories, and select the best fit through fitness and difference tests. As the classification of the model increases, the AIC, BIC, and aBIC values continue to decrease, and the entropy value approaches 1. LMR and BLRT reach significant levels (both *P* < 0.05), indicating that the model has reached the optimal level.

The mean standard deviation was used to describe the measurement data, frequency and percentages were used for counting data, and the chi-square test or nonparametric test was used for comparison between multiple groups. Harman single factor test method for common method bias testing. Unordered multicategorical logistic regression was used to analyze the factors influencing the perceived categories of decent work among nursing students. The social demographic characteristics that may have an impact on the results are included as covariates in the model, and finally, the likelihood ratio test is synchronously conducted to test whether the independent variable is meaningful. The test level is 0.05, and the difference between the two tests is statistically significant with *P* < 0.05.

### Ethical review and consent participation

The Harbin Medical University Second Hospital Ethics Committee authorized this investigation (KY2022-311). When setting up our online questionnaire link, we placed the informed consent form on the first page of the questionnaire. After participants click on the link to enter, they will first see the informed consent form. Only after participants confirm their informed consent can they complete the survey questionnaire. And at the top of the questionnaire, inform participants of the purpose and methods of this study, ensure their voluntary and anonymous participation, and ensure the confidentiality of their answers. In addition, the questionnaire specifically notes the name and contact information of the data management personnel at the end and prompts participants. If there are any problems during the survey or if you want to withdraw, you can contact them at any time. All methods are carried out in accordance with relevant guidelines and regulations.

## Results

### Harman single factor test method

The results showed that there were a total of 12 factors with feature roots greater than 1. The first factor explained a variance of 25.221%, which is less than the critical standard of 40% [35], indicating that there is no serious common method bias in the research data. This result has been supplemented in the article, increasing the scientific nature of data collection and processing.

### General information

82.2% of participants (*n* = 630) were female, 90.3% were specialized students, 84.4% were sophomores, and 55.1% were only children. 55.1% were from rural areas; 61.4% occasionally participated in college activities each month; 59.7% came from a voluntarily chosen nursing program; 79.2% had not participated in clinical practice at the time of this study; 41.9% had a monthly family economic income of less than 3000 yuan; and 56.5% were surrounded by admired nursing seniors. Reference Table 1.

**Table 1:** General data statistics for nursing students (*n* = 630)

Item	Classification	Number	Constituent ratio
Gender			
	Male	112	17.8
	Female	518	82.2
Educational background			
	Junior college education	569	90.3
	Undergraduate course	61	9.7
Grade			
	Sophomore	532	84.4
	Junior	58	9.2
	Senior	40	6.3
Be the only child or not			
	Yes	347	55.1
	No	283	44.9
Origin of student			
	Rural area	347	55.1
	Urban area	283	44.9
Monthly active participation in various activities organized by the college			
	Regularly attend(more than two times per month)	114	18.1
	Occasionally attend(one or two times per month)	387	61.4
	Never	129	20.5
Whether to choose nursing voluntarily			
	Yes	376	59.7
	No	254	40.3
Duration of clinical practice			
	Have not yet participated	499	79.2
	Within one month	21	3.3
	1–3 months	22	3.5
	3-6 months	32	5.1
	More than 6 months	56	8.9
Average monthly household income (in yuan)			
	< 3000	264	41.9
	3000–5000	244	38.7
	> 5000	122	19.4
If you have someone you admire in nursing			
	Yes	356	56.5
	No	274	43.5

### Scores of each scale

Total occupational identity (62.15 ± 11.978), self-regulation fatigue (42.51 ± 9.746), and perception of decent work (49.64 ± 6.250) were the mean scores of nursing students. For more information, see Table 2.

**Table 2:** Score statistics of each scale (*n* = 630)

Variable	Minimum	Maximum	Average value	Standard deviation	Skewness	Kurtosis
Vocational self-concept dimension	6	30	22.63	5.009	-0.350	0.173
Benefits of retention and turnover risk dimension	4	20	14.11	3.380	-0.057	-0.024
Social comparison and self-reflection dimension	3	15	11.23	2.414	-0.097	-0.150
Autonomy in career choice dimension	3	10	6.66	1.369	1.066	0.748
Social persuasion dimension	2	10	7.52	1.642	-0.184	-0.239
Total score on the Occupational Identity scale	21	85	62.15	11.978	-0.076	-0.164
Cognitive controls dimension	6	28	16.67	2.951	-0.431	1.073
Behavior control dimension	5	25	12.81	4.770	0.318	-0.049
Emotional control dimension	5	25	13.03	3.541	-0.499	0.236
Total score on the self-regulatory fatigue	20	74	42.51	9.746	-0.323	-0.192
Work return dimension	4	20	11.70	2.541	-0.209	1.507
Position dimension	3	15	8.44	1.996	-0.095	2.388
Career development dimension	3	15	9.77	1.654	0.715	3.284
Professional identity dimension	3	15	9.62	2.090	0.240	2.154
work atmosphere dimension	3	15	10.12	2.017	0.577	1.156
Total score on the decent work perception	20	75	49.64	6.250	0.439	2.641

### Latent profile analysis of nursing students' perceptions of decent work

In this survey, the five dimensions of the Decent Work Perception Scale were utilized as indicators, and the types of nursing students' decent work perceptions were categorized into 1, 2, 3, and 4 categories for an estimate of model fitting. The results demonstrated that the AIC and BIC values fell as the number of categories climbed from 1 to 4, and that the LMR and BLRT achieved a significant level (both *p* < 0.05) when retained in 2 categories as well as when retained in 3 categories. Nonetheless, the category 2 entropy value of 0.86 is less than category 3's value of 0.93. In the model fit index, "the closer the entropy value is to 1, the higher the classification accuracy," hence category 3 has a higher classification accuracy than category 2, and model 3 is therefore superior to model 2. The LMR did not reach a significant level (*P* > 0.05) when reduced to four categories, showing that model 3 was superior to model 4, and hence model 3 was the best model. See Table 3.

**Table 3:** Latent profile analysis of nursing students' decent work attitudes (*n* = 630)

Model	LL	AIC	BIC	aBIC	LMR	BLRT	Enrtopy	Class probability
1	-7108.362	14236.724	14281.181	14249.433	-	-	-	-
2	-6781.395	13594.791	13665.922	13615.124	0.0000	0.0000	0.860	0.72540/ 0.27460
3	-6536.761	13117.521	13215.327	13145.480	0.0228	0.0000	0.931	0.04762/ 0.25873/ 0.69365
4	-6405.491	12866.981	12991.461	12902.564	0.1603	0.0000	0.939	0.25079/ 0.04603/ 0.04603/ 0.65714

### Naming of potential categories of nursing students' perception of decent work

**Fig. 1:**
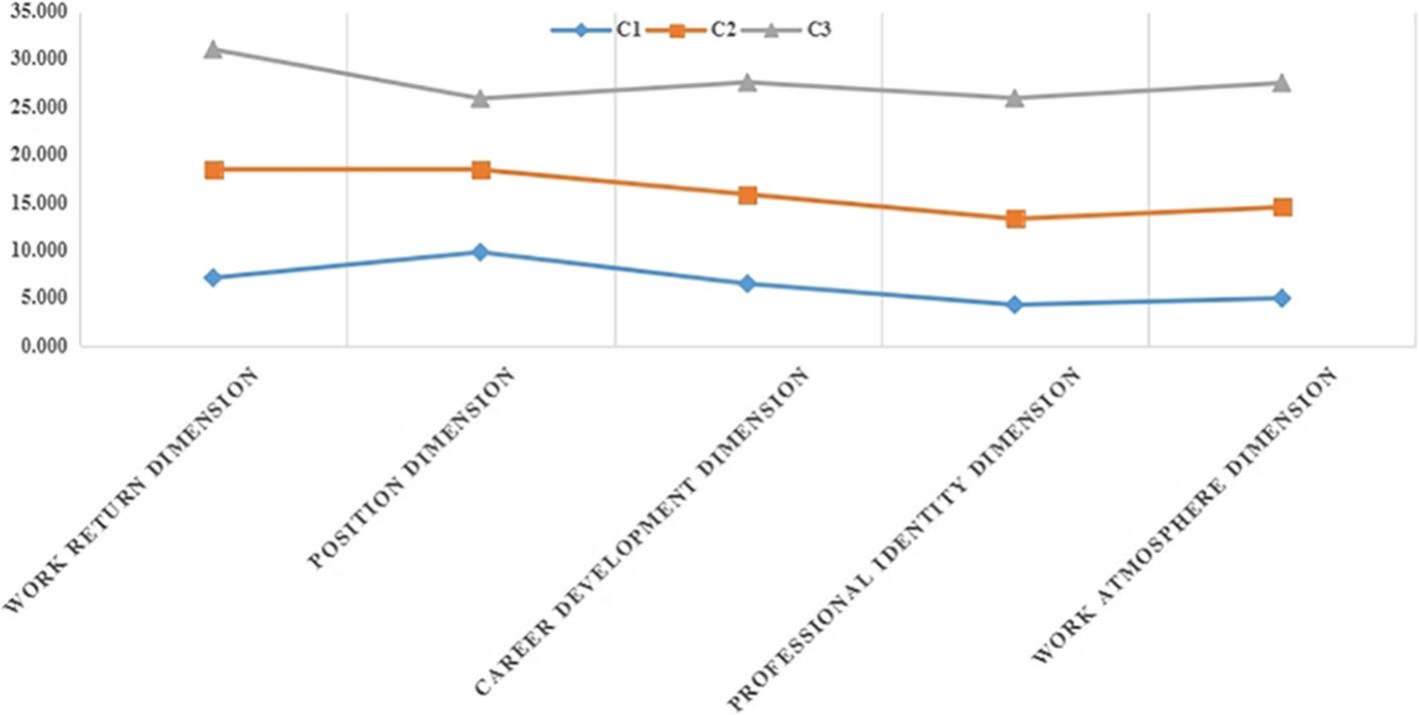
Nursing students' decent work subcategories for the 3-level model

The 3 categories were used to plot nursing students' scores on the 5 dimensions of decent work perceptions. The dimensions were horizontal, and the scores were vertical. Figure 1 shows nursing students' decent work subcategories for the 3-level model.

According to the explicit characteristics of each dimension of the scale, each category was named. The nursing students of the first perceived type scored at a lower level in each dimension (with the reverse score of the job position dimension also being lower), indicating that nursing students have a lower level of perception of future work salary, work atmosphere, career development, and other aspects. Therefore, C1 was named the "Low Level Decent Work Perception Group", consisting of 30 people, accounting for 4.76% of the total sample. The scores of nursing students in the second type of perception are at a moderate level in all dimensions. Therefore, C2 was named the "Perceived Group of Moderate Level Decent Work", with a total of 437 people, accounting for 69.37% of the total sample. The scores of nursing students in the third type of perception are above average in all dimensions, indicating that nursing students are full of aspirations and expectations for clinical work benefits, environment, future career development, and other aspects. Therefore, C3 was named the "High Level Decent Work Perception Group", consisting of 163 people, accounting for 25.87% of the total sample.

### Intergroup comparison of nursing students' perceptions of decent work

There were statistically significant differences in the total score of nursing students' perception of decent work and scores of all dimensions among the "Low Level Decent Work Perception Group (C1)," "Perceived Group of Moderate Level Decent Work (C2)," and "High Level Decent Work Perception Group (C3)" (all *P* < 0.001), as shown in Table 4 for details.

**Table 4:** Comparison of differences among nursing students' perception of decent work (*n* = 630)

Item	C1	C2	C3	F	*P*
Work return dimension	7.17 ± 3.46	11.35 ± 2.060	13.54 ± 3.033	103.842	< 0.001
Position dimension	9.83 ± 4.145	8.65 ± 1.586	7.45 ± 2.727	26.561	< 0.001
Career development dimension	6.50 ± 2.874	9.35 ± 1.066	11.74 ± 1.774	258.333	< 0.001
Professional identity dimension	4.23 ± 1.695	8.99 ± 1.219	12.64 ± 1.659	672.105	< 0.001
work atmosphere dimension	4.97 ± 1.866	9.51 ± 1.194	13.02 ± 1.559	653.717	< 0.001
Total score on the decent work perception	32.70 ± 5.943	47.86 ± 3.538	58.39 ± 5.550	613.097	< 0.001

### Univariate examination of potential categories of nursing students' perceptions of their profession

There were statistically significant differences (*P* < 0.05) between the three groups in the frequency of monthly active participation in various activities organized by the college, the reasons for choosing the nursing profession, the average monthly family economic income, the number of nursing seniors around whom admiration exists, occupational identity, and self-regulation fatigue. Other general information showed no statistically significant differences (*P* > 0.05). See Table 5.

**Table 5:** Univariate analysis of potential categories of decent work perception among nursing students (*n* = 630)

Item	Classification	Low Level Decent Work Perception Group (*n* = 30)	Perceived Group of Moderate Level Decent Work (*n* = 437)	High Level Decent Work Perception Group (*n* = 163)	χ^2^/F	*P* value
Gender					1.125	0.570
	Male	6(5.4%)	73(65.2%)	33(29.5%)		
	Female	24(4.6%)	364(70.3%)	130(25.1%)		
Educational background					3.573	0.179
	Junior college education	30(5.3%)	394(69.2%)	145(25.5%)		
	Undergraduate course	0(0.0%)	43(70.5%)	18(29.5%)		
Grade					6.519	0.143
	Sophomore	27(5.1%)	365(68.6%)	140(26.3%)		
	Junior	0(0.0%)	41(70.7%)	17(29.3%)		
	Senior	3(7.5%)	31(77.5%)	6(15.0%)		
Be the only child or not					1.018	0.601
	Yes	19(5.5%)	241(69.5%)	87(25.1%)		
	No	11(3.9%)	196(69.3%)	76(26.9%)		
Origin of student					3.743	0.156
	Rural area	12(3.5%)	239(68.9%)	96(27.7%)		
	Urban area	18(6.4%)	198(70.0%)	67(23.7%)		
Monthly active participation in various activities organized by the college					28.310	0.000
	Regularly attend (More than two times per month)	9(7.0%)	94(72.9%)	26(20.2%)		
	Occasionally attend(one or two times per month)	14(3.6%)	277(71.6%)	96(24.8%)		
	Never	7(6.1%)	66(57.9%)	41(36.0%)		
Whether to choose nursing voluntarily					11.660	0.003
	Yes	10(2.7%)	258(68.6%)	108(28.7%)		
	No	20(7.9%)	179(70.5%)	55(21.7%)		
Duration of clinical practice					8.005	0.370
	Have not yet participated	24(4.8%)	354(70.9%)	121(24.2%)		
	Within one month	2(9.5%)	13(61.9%)	6(28.6%)		
	1–3 months	2(9.1%)	14(63.6%)	6(27.3%)		
	3-6 months	0(0.0%)	22(68.8%)	10(31.3%)		
	More than 6 months	2(3.6%)	34(60.7%)	20(35.7%)		
Average monthly household income /Yuan					11.255	0.024
	< 3000	17(6.4%)	188(71.2%)	59(22.3%)		
	3000–5000	6(2.5%)	176(72.1%)	62(25.4%)		
	> 5000	7(5.7%)	73(59.8%)	42(34.4%)		
If you have someone you admire in nursing					28.310	0.000
	Yes	10(2.8%)	227(63.8%)	119(33.4%)		
	No	20(7.3%)	210(76.6%)	44(16.1%)		
Total score on the self-regulatory fatigue		45.60 ± 13.114	41.11 ± 11.762	42.79 ± 9.062	3.173	0.043
Total score on the occupational identity		54.50 ± 18.044	60.49 ± 11.454	69.50 ± 10.393	43.501	0.000

### Multimodal logistic regression study of nursing student opinions of decent work categories

According to the parallelism test result *P* = 0.000, the null hypothesis was rejected and unordered multi-classification Logistic analysis was adopted. Three variables, namely the C1 low level of decent work perception group, the C2 medium level of decent work perception group, and the C3 high level of decent work perception group, were used as outcome variables. The independent variable assignment method is shown in Table 6. The covariates included gender, education level, grade, whether they are only children, place of origin, and time of participating in clinical internships, and the influence of confounding variables on the results was excluded. Using "C3" as the reference group, compare the differences in the characteristics of nursing students' perceived types of decent work.

**Table 6:** Assignment of independent variables

Variable	Assignment
Monthly active participation in various activities organized by the college	Never = 1 Occasionally attend = 2; Regularly attend = 3(reference);
Whether to choose nursing voluntarily	No = 1; Yes = 2(reference);
If you have someone you admire in nursing	No = 1; yes = 2(reference);
Average monthly household income	< 3000 = 1; 3000–5000 = 2; > 5000(reference);
Total score on the occupational identity	Original entry
Total score on the self-regulatory fatigue	Original entry

The results showed that the statistically significant indicators were involuntary choice of nursing major, low average monthly family economic income, occupational identity, and no respect for nursing seniors (all *P* < 0.05). The above statistically significant variables are all between C2 and C3, and there is no significant difference between C1 and C3. The specific description is that, with "C3" as a reference, the involuntary selection of nursing majors (0R = 1.691, *P* = 0.001), no respected nursing seniors (0R = 0.650, *P* = 0.008), the average monthly family economic income is less than 3000 yuan (0R = 0.419, *P* = 0.000), the average monthly family economic income is between 3000–5000 yuan (0R = 0.591, *P* = 0.003), professional identity (0R = 1.079, *P* = 0.000) is classified as C2, see Table 7 for details.

**Table 7:** Multiple logistic regression of three potential categories of factors influencing the perception of decent work for nursing students (*n* = 630)

Variables	B	SE	Waldχ2	P	OR	(95%CI)	
						**Lower limit**	**Upper limit**
**C1 Low Level Decent Perception Group VS C3 High Level Decent Work Perception Group**							
Total score on the occupational identity	-0.023	0.018	1.645	0.200	0.977	0.942	1.012
Total score on the self-regulatory fatigue	0.026	0.023	1.283	0.257	1.027	0.981	1.074
Monthly active participation in various activities organized by the college							
Never	0.874	0.584	2.244	0.134	2.397	0.764	7.524
Occasionally attend	-0.119	0.458	0.067	0.796	0.888	0.362	2.181
Regularly attend(reference)							
Whether to choose nursing voluntarily							
No	0.709	0.459	2.392	0.122	2.032	0.827	4.993
Yes (reference)							
If you have someone you admire in nursing							
No	0.296	0.468	0.399	0.527	1.344	0.537	3.362
Yes (reference)							
Average monthly household income /Yuan							
< 3000	0.149	0.497	0.090	0.764	1.161	0.438	3.078
3000–5000	-0.775	0.587	1.744	0.187	0.461	0.146	1.455
> 5000(reference)							
**C2 Medium Level Decent Perception Group VS C3 High Decent Work Perception Group**							
Total score on the occupational identity	0.076	0.007	108.908	0.000	1.079	1.064	1.094
Total score on the self-regulatory fatigue	0.012	0.007	2.931	0.087	1.012	0.998	1.026
Monthly active participation in various activities organized by the college							
Never	0.121	0.236	0.263	0.608	1.128	0.711	1.791
Occasionally attend	-0.198	0.191	1.070	0.301	0.821	0.564	1.193
Regularly attend(reference)							
Whether to choose nursing voluntarily							
No	0.525	0.159	10.940	0.001	1.691	1.239	2.308
Yes (reference)							
If you have someone you admire in nursing							
No	-0.431	0.163	6.990	0.008	0.650	0.472	0.895
Yes (reference)							
Average monthly household income /Yuan							
< 3000	-0.870	0.188	21.493	0.000	0.419	0.290	0.605
3000–5000	-0.526	0.179	8.660	0.003	0.591	0.416	0.839
> 5000(reference)							

Take C3 as reference

In addition, the results of the likelihood ratio test are shown in Table 8, further verifying the influencing factors of the potential categories of decent work perception among nursing students. The results also show that there is statistical significance in choosing nursing majors, whether there are nursing seniors who are respected, the average monthly income of the family economy, and occupational identity (all *p* < 0.05).

**Table 8:** Validation of Likelihood Ratio (L.R.) test results

Effect	Simplification of Model Fitting Conditions -2 Logarithmic Likelihood of the Model	χ^2^	*P*
Total score on the occupational identity	1814.957	127.269	0.000
Total score on the self-regulatory fatigue	1691.722	4.033	0.133
Monthly active participation in various activities organized by the college	1694.839	7.151	0.128
Whether to choose nursing voluntarily	1700.653	12.965	0.002
If you have someone you admire in nursing	1695.401	7.713	0.021
Average monthly household income /Yuan	1713.442	25.754	0.000

## Discussion

### Nursing students' overall perception of decent work is at a moderate level

The perception score of decent work for nursing students was(49.64 ± 6.250) (total score: 80), indicating a moderate perception. It falls below the estimates of Chinese academics such as Jiang Hanying [36]. Her study of nurses in general hospitals yielded a score of (50.94 ± 12.61), and unlike registered nurses, nursing students are transitioning to clinical staff. The level of decent work perceptions among nursing students in this survey suggests that there may be a gap between the career aspirations of nursing students in their school studies and the career reality in hospitals, leading to a diminished sense of decency.

### Three categories of perceived decent work for nursing students

In this study, nursing students' perceptions of decent work were identified into three subgroups: low-level decent work perception group", medium-level decent work perception group", and high-level decent work perception group".

The "low-level decent work perception group" accounts for 4.76%. Based on their response scores, this type of nursing student has a lower perception of future job returns, career development, career identity, work atmosphere, and other aspects. This can be analyzed from two different angles. Firstly, research indicates that the degree of stress among Chinese nursing students is relatively high [37]. Fear of making errors, problems related to death and near-death experiences, witnessing pain and suffering, interpersonal problems with clinical faculty and nursing staff, communication with physicians, unfamiliarity with the hospital environment, and numerous other stressors can induce negative emotions in nursing students during clinical practice, thereby lowering their career expectations [38–40]. The lowest perceived workplace score (8.44 ± 1.996) of nursing students in this study (total score of 15) reflects their impressions of a high-pressure clinical work environment. According to a research study of nurses conducted by Zhang et al. [41], stress influences nurses' judgments of decent work, and the more stressful their jobs are, the lower their opinions of decent work.

The "medium level decent work perception group" accounts for 69.37%, and more than half of nursing students have a moderate level of perception of future decent work. indicating that their perceived level of decent work is at a moderate level. This may be related to the insufficient guidance on vocational development education in China at present [42]. The International Association of Educational and Vocational Guidance (IAEVG) highlights that "good educational and vocational guidance and counseling can assist individuals in recognizing their strengths and potential and enable them to plan measures to develop important skills. Yet, the current absence of adequate career guidance in colleges and universities hinders nursing students' awareness of career-related information and competencies.

The "high level decent work perception group" accounts for 25.87%, and this type of nursing student has a high level of perception of future decent work, with a higher score in the work return dimension. Research has pointed out that job return is an important influencing factor for the perception of decent work among the new generation of employees [43]. This suggests that in the future, organizations should establish a competitive and motivational job return system to enhance the sense of decent work return among the new generation of employees.

### Differences in perceived types of decent work among nursing students in terms of demographic and occupational identity

In this study, using "C3" as a reference, the greater the likelihood of involuntarily choosing a nursing major, having no respect for nursing seniors, having a low average monthly income from a family, and having a professional identity classified as C2.

To begin, our research found that, similar to the findings of Kim et al. [44], nursing students have low household income and relatively low perceptions of decent work compared to high perceptions of decent work. In their study, Lee et al. [45] found that if a family's socioeconomic level is low, parents and neighbors will not have great expectations for their children's careers, and they will typically make career decisions to obtain employment. A significant proportion of nursing students in China are from middle- and low-income socioeconomic backgrounds [46]. For them, solid employment is necessary to overcome economic challenges and even poverty [6, 47]. In other words, nursing students with financial difficulties have a strong desire for employment but a low opinion of decent work, whereas nursing students with adequate financial resources find jobs that are more consistent with their standards and have a higher perception of decent work [48].

Another interesting finding is that, when compared to the perception of a high level of decent work, the chance of nursing students who pursue nursing majors involuntarily entering a perception of a medium level of decent work in the future is 1.691, which is comparable to the findings of Zhao et al. [28]. Bench and Muntane [49] hypothesized in their study that those who "voluntarily" accept employment are more likely to feel job dissatisfaction and stress than those who "involuntarily" accept employment. This may explain the "involuntary" nature of nursing students who are exposed to professional adjustment experiences and why the choice of nursing majors is involuntary. These "involuntary" nursing students are involved in the nursing sector because they are forced to transfer to the nursing major due to poor grades on the college entrance exam or they choose the nursing major against their will due to pressure from their relatives and friends. Such students lack an in-depth understanding of the nursing sector and lack excitement for the nursing industry, which diminishes to some extent the perception of nursing students' decent work [50, 51].

Furthermore, it should be noted that a considerable proportion of the students polled in our study are in the early phases of practical training, with the majority lacking genuine clinical internship experience. Their job expectations and understanding may not be completely formed, or they may be based primarily on theoretical knowledge rather than real experience. Student value counseling is critical at this time since research has shown that values can alter the process of individual perception, matching people's perception of external stimuli with their value system [52]. This is consistent our the study's findings. We discovered that "non-respected nursing seniors" entering the middle level have a 0.650 times greater perception of decent work than "respected nursing seniors". Respected nursing seniors have a relatively high opinion of decent work, which is consistent with Li Feifei et al.'s research findings [53]. Poorchangizi et al. [54] and [55] noted that students can increase their awareness of the nursing profession through their educators or non-teacher nurses. This demonstrates that the power of example is a spiritual meal that cannot be undervalued, as it can guide the way for the development and evolution of pupils and can even rise to the level of value guidance.

One revelation deserves our attention. In this study, occupational identity had a significant impact on nursing students' judgments of decent work, which is consistent with research by Hu et al. [56]. Occupational identity reflects how nursing students perceive the specialty of nursing. The research indicates that decent work is a form of recognition and pride among social workers for their own occupation, and that these factors also reflect occupational safety, occupational attribution, and occupational value to a certain extent and are significant sources of occupational identity among social workers. Consequently, when social workers have a strong sense of occupational decency, their occupational identity will be elevated, and decent work can considerably and favorably enhance occupational identity [56].

Finally, we went into detail about why, in this study, variables like involuntary choice of nursing major, lack of respect for nursing seniors, low average monthly family income, and occupational identity had no impact between C1 and C3. The nursing students in the low-level perceived decent work group in this study scored at a low level in the dimensions of work return (7.17 ± 3.465), job position (9.83 ± 4.145), career development (6.50 ± 2.874), career recognition (4.23 ± 1.695), work atmosphere (4.97 ± 1.866), and total score (32.70 ± 5.943). The average score of the items ranged from 1 to 2, indicating a complete or lesser degree of recognition. For example, they believe that the future pay from this job will not meet their living expenses, that the future workload will be extremely demanding, and that the position or rank promotion will fall short of their expectations after a few years of employment. The assessment is low, unfavorable, and unfavorable overall.

The reason may be due to the influence of the concept of "low social status of nurses," coupled with the heavy workload and high risks, as well as the increasing awareness of patient rights protection, tense nurse-patient relationships, and frequent incidents of hospital violence in the work environment, which leads to their dislike or difficulty liking this profession and lower satisfaction with the nursing profession [57]. Therefore, regardless of factors such as family economic status and the worship of nursing seniors, it is difficult to reverse the attitude of this group of nursing students towards the nursing profession. As research shows, 24.9% of nursing students believe that work intensity is high, requirements are high, and night shifts are frequent; 12.1% of nursing students believe that only one job is needed; 7.1% think it is not easy to find employment [58], and 35.5% of nursing students believe that the nursing industry environment is very and relatively unsafe [59]. This also leads to some nursing students switching majors or graduating without engaging in nursing work. A recent survey conducted in 31 provinces (autonomous regions, municipalities directly under the central government) in China targeting 9156 nursing students showed that 67.9% of nursing students chose to work in the nursing profession, 6.3% chose to work in non-nursing professions, and 25.8% chose not to work temporarily, which has greatly caused the loss and waste of talent resources [58]. This also suggests that universities and relevant departments attach great importance to the level of perceived dignity among nursing students, especially for nursing students with low levels of perceived decent work. It is even more important to coordinate the top-level design and employment guidance of schools, industries, and individual nursing students in order to stabilize the reserve force of the nursing team.

### Advantages and limitations

One of the main benefits of this study is that it looked at how nursing students in different subgroups classify and describe decent work.According to our knowledge, this is the first study to include nursing students. In addition, this study indicates that nursing students' perceptions of their decent work are moderate and warrant attention. In addition, this is the first study to be discussed. Moreover, this study addresses the career-guiding method of nursing students' perceptions of decent work. For instance, pay attention to the guiding perspective of decent work perception, boost professional publicity, strengthen advice on role models, and increase occupational identity. In addition, it is important to note that this study included the nursing student variable of self-regulating fatigue. Although it was confirmed that there was no difference between self-regulating fatigue and decent work perception among nursing students in the subgroup of decent work perception, the correlation between self-regulating fatigue and decent work perception among nursing students could not be ruled out. The correlation between self-regulating weariness and perceptions of decent work was corroborated by additional SPSS analysis, which revealed a correlation between the two variables. Refer to the additional data for the results.

Undoubtedly, every study must have constraints. The following highlights the main limitations of this study: Firstly, we try our best to consider all potential screening criteria, but it may not cover all potential issues, which may result in us not being able to ensure that all students meet the requirements for filling out the questionnaire. Secondly, this study did not cover all nursing students in grades one to four, which may lead to a decrease in data persuasiveness. In the future, it is recommended to add an analysis on the perception of decent work among first-year nursing students. Thirdly, this study uses a convenient sampling method, which may result in an unrepresentative sample of the entire student population, limiting the findings' generalizability. In the future, a more scientific sampling procedure is anticipated. Fourthly, our survey questionnaire is provided in the form of self-reporting, and the resulting memory bias or social expectation bias is unlikely to be avoided. As said, many of the polled students are in the early phases of practical training, so their job expectations and comprehension may be based on theory rather than practice. This also recommends that we study nursing students with internship experience and their views on decent labor. Finally, because of the cross-sectional design, it is impossible to guarantee the causal link between variables. Future research can make up for this by gathering longitudinal data to strengthen the persuasiveness of the conclusions.

### Research guidance and significance

The results of this study will help colleges, universities, and relevant departments put the idea of decent work at the top of their lists and figure out how to teach people. First, when it comes to career guidance, colleges and universities should pay more attention to helping nursing students figure out what they want, really understand and accept their majors, and pay special attention to helping nursing students from low-income families figure out what they think is decent work. Second, in terms of vocational publicity, we should strengthen the professional publicity in enrollment and the guidance and education for students after applying for the examination, strengthen the understanding of nursing students' majors, and improve the professional recognition of nursing students. Thirdly, in the aspect of model setting and value guidance, it is suggested that colleges and universities should strengthen communication and cooperation with teaching teachers and student families and infiltrate model guidance and value guidance into professional education, practical education, ideological and political education, and other aspects to improve nursing students' sense of decent work. Fourth, in the aspect of career identity guidance, at the key stages of students' lives (such as enrollment, daily classes, clinical practice, and other links), professional career planning, future employment prospects, and other related special lectures increase the occupational identity of nursing students, reduce the level of role ambiguity, and improve the sense of decent work.

## Summary

This study employs latent profile analysis to uncover the category characteristics and influencing elements of Chinese nursing students' perceptions of decent work, taking into account the nursing students' individual differences and professional qualities. The research results provide a theoretical foundation for colleges and universities and relevant departments to conduct vocational guidance for students, in addition to new ideas and insights for conducting vocational guidance from the perspective of decent work perception, which is conducive to implementing targeted intervention programs for different types of nursing students.

## Data Availability

The data sets for this study are available from the appropriate authors under reasonable conditions. Contact Zhang Yuhuan, email 2802262584@qq.com.
